# Madness and Marriage

**Published:** 1919-08-09

**Authors:** 


### MADNESS AND MARRIAGE.

Cynics may say that the two are inseparable, and no doubt with some people the second of these does raise a presumption of the first; but there are occasions when the first so far from conducing to the second, as is perhaps usual, is alleged as a reason for nullifying it. Even in such cases, however, the first has been the cause of the second. A case in which the connection between the two has been discussed in a Court of Law has just been decided by Mr. Justice Shearman, in the Probate and Divorce Division of the High Court. A gentleman, who had been certified as insane on and off for about ten years, was detained as a lunatic in the licensed house at Ticehurst. While a patient in this establishment, he became enamoured of a housemaid employed there, who was dismissed for undue familiarity with the patient. The patient contrived to escape, and married the housemaid, upon which the son of the bridegroom brought an action as guardian *ad litem* of his father for the nullification of the marriage, and succeeded in obtaining a decree.

Suits for nullity of marriage on the grounds of insanity are very rare. As far as we know, only one such case has been tried since the Durham case in the year 1884. In the Durham case, strong as the evidence was, the plaintiff was not successful, but in the subsequent case to which we allude, a decree was obtained. This case was remarkable from the fact that the husband, who was the defendant in the suit, was a paranoiac, and was not recognised as being insane until some time after the marriage, yet it was proved at the trial that his insanity existed unrecognised at the time of the marriage, and was of such a character and degree as to disqualify him from entering into the contract. What makes the case stronger was that at the time of the trial there was a child of the marriage. In the case just tried by Mr. Justice Shearman, there could be little doubt as to the madness of the bridegroom at the time of the marriage, for he had at that time but recently escaped from a lunatic asylum, in which he was duly detained under certificates and a magistrate's order. He was not, however, a lunatic so found by inquisition.

It would be a mistake to suppose that a lunatic is as such disqualified from entering into a contract. He can certainly enter into ordinary business contracts, and such contracts have been upheld by the courts in spite of the lunacy of the contractor. In order to avoid a contract on the ground of insanity, it is necessary, however, that the madness should be of such a nature that it would be supposed to have influenced his mind in entering into the contract. The contract of marriage is of a more sacred character than that of ordinary business contracts; it is more binding; and the court has shown itself extremely reluctant to dissolve a marriage on the ground of the insanity of the bridegroom or bride at the time the contract was made. As with other contracts, the madness must be of such a character as to influence the mind of the contractor in entering into it, or the contract will be upheld by the court. In this case the judge was of the opinion that the madness was of that character. He held that the petitioner was suffering from "erotic mania," a term antiquated and obsolete in medicine, but still, it would appear, recognised in law, and the madness being of this kind, the learned judge "was satisfied that the petitioner was influenced by this erotic mania, and that he had not the judgment of a sane man when he went through the ceremony of marriage with the respondent, nor did he recognise and appreciate the responsibilities of that particular marriage." That is to say, he was not only generally incompetent to marry, but also he was incompetent to contract the particular marriage under consideration.

The case is noteworthy for its rarity, and also for the fact that the judgment rested upon common law grounds, and not upon the provisions of any statute, though statutes were referred to by the learned judge. It would appear from this, as from previous judgments, that a lunatic may not only enter into a binding contract of business, but that he may also enter into a binding contract of marriage, provided that his madness is not of such a character as to influence him in the performance of the very act itself; that is to say of the particular marriage that he is contracting.

